# Comparison between Quality Traits of Solar-Dried and Freeze-Dried *Origanum syriacum* L. (Za’atar)

**DOI:** 10.3390/plants11091110

**Published:** 2022-04-20

**Authors:** Reem Abu Alwafa, Samer Mudalal, Faisal Shraim, Gianluigi Mauriello

**Affiliations:** 1 Department of Agricultural Science, University of Naples Federico II, 80055 Portici, NA, Italy; abualwafa.reem@gmail.com (R.A.A.); giamauri@unina.it (G.M.); 2 Department of Nutrition and Food Technology, Faculty of Agriculture and Veterinary Medicine, An-Najah National University, Nablus P.O. Box 7, Palestine; 3 Department of Plant Production and Protection, Faculty of Agriculture, An-Najah National University, Nablus P.O. Box 707, Palestine; fshraim@najah.edu

**Keywords:** freeze drying, solar drying, sensory, chemical composition, za’atar

## Abstract

There is growing interest in *Origanum syriacum* due to attractive culinary applications and functional properties. Solar drying is the most common conventional method for drying *Origanum syriacum L.* (Za’atar) in Mediterranean region. This study aimed to evaluate the quality traits of solar dried za’atar in comparison to freeze dried za’atar. Proximate composition (moisture, protein, fat, fiber, carbohydrates, and ash), color index (L*a*b*), macro and microstructure, sensory, and microbiological characteristics were evaluated. Solar dried za’atar exhibited significantly lower fat content (1.10 vs. 1.64%, *p* < 0.05) than freeze dried za’atar. Solar drying led to severe changes in color values compared to freeze drying. Solar drying exhibited loss in the structural integrity and leave hairs more than freeze drying. Moreover, freeze-dried za’atar exhibited significantly lower total aerobic count (4.92 vs. 5.23, *p* < 0.05) and yeasts and mold count (4.59 vs. 5.36, *p* < 0.05) than solar-dried za’atar. Panelists were able significantly to differentiate between solar- and freeze dried za’atar. Freeze-dried za’atar had significantly lower hedonic score of color, odor, taste, and overall acceptance than solar dried za’atar. In conclusion, using freeze drying achieved good improvements in some quality traits for za’atar while solar dried za’atar showed better sensory perception.

## 1. Introduction

There are different species of oregano (genera: *Origanum*) distributed over the world that belong to Lamiaceae family [1]. Lamiaceae family contains more than 260 genera and 7000 species [2]. One the most common species of oregano is *Origanum syriacum L*. that is usually cultivated in Mediterranean, western Asia, and southern Europe [3] and it has high significant economic value in these regions [4,5]. There are common names for *Origanum syriacum L.* such as *Lebanese oregano*, za’atar”, *Bible hyssop*, and *Syrian oregano* [3].

There are many culinary applications for “za’atar”. It is usually used as dried mix (salt, seasons, and sesame). This mix is usually used with olive to form paste and this paste spreads over dough then baked similar to pizza and it is called “Man’ousheh” in Arabic [6]. In addition, za’atar is used as herbal tea as well as seasoning in cooking. There are several commercial forms for za’atar such as fresh za’atar, dried za’atar, and essential oil herb.

The quality of *O. syriacum* is affected by several pre- and post-harvesting factors. It was found that air drying technique for *O. syriacum* was more effective in retention of aroma, flavor, and color in respect to oven- and freeze-drying [7]. Drying temperature had critical effect on chlorophyll and carotenoids contents of *O. syriacum*. High drying temperature led to higher destruction chlorophyll and carotenoids contents in comparison to low drying temperature [8]. 

*O. syriacum* is very rich in several functional, nutritional, aromas, and flavoring compounds. Accordingly, during drying of *O. syriacum*; the loss of color, taste, flavor, appearance, and chemical composition should be minimized [9]. The most common method that is usually used to dry *O. syriacum* in Mediterranean region is solar drying. There were many limitations for solar drying such as the prolonged drying time, inadequacy of drying, and the effect of the weather [10]. Solar drying is usually carried out under the direct sunlight which leads to more loss in essential oils. Evaporation of essential oils increased sharply at temperature more than 30–35 °C. Therefore, to reduce the loss of essential oils, temperature should be controlled [7,11].

It was found that increased soil moisture decreased the content of dry matter in *O. syriacum*. The irrigation levels did not show any effect on the content of thymol and carvacrol in the essential oil of *O. syriacum* [7]. Air-dried *O. syriacum* exhibited strong, bitter, pungent, warm, and astringent flavors with camphor-like, strong, and pungent aromas. Freeze-dried *O. syriacum* had high content sesquiterpenes that resulted in undesired earthy and musty aroma. The loss of carvacrol in the essential oil of *O. syriacum* was increased by the application of oven and freeze-drying. The content of thymol and carvacrol and essential oil yield were varied according to geographical regions [12,13,14]. 

There is no enough knowledge about the effect processing conditions on the quality traits of za’atar. Accordingly, further investigations are needed to have full understanding about optimum processing conditions to obtain minimally processed dried za’atar. The aim of this study is to evaluate the effect of freeze drying as alternative technique for solar drying on quality traits of *O. syriacum*.

## 2. Materials and Methods

### 2.1. Samples Collection and Preparation

The sample weight of *O. syriacum* that has been employed in this study was about 4 kg as fresh weight. It has been harvested from field cultivated in traditional way with irrigation system in Tulkarem, Palestine in August 2020. The leaves of za’atar were manually separated and the abnormal leaves (containing darken areas or yellowness) were excluded. 

The quantity of *O. syriacum* was divided into 3 groups. The first group was kept as it is (fresh) without any treatments. The second group was subjected to freeze drying by using BENCHTOP Manifold Freeze Dryer from Millrock Technology Inc. (Kingston, NY, USA) at −80 °C and 50–200 mbar for about 24 h. The third group was subjected to solar drying as farmers do in Palestine and the weather conditions during solar dying are shown in Table 1. In conventional solar drying, the *O. syriacum* leaves with stems were spread over plastic roll sheet on the ground and left for few days until it gets dry. 

### 2.2. Proximate Chemical Analysis 

Proximate analysis (moisture, proteins, fat, ash, and fibre) has been determined for all groups according to AOAC [16] and carbohydrate content was calculated by difference. Moisture content was measured by weight loss at 105 °C for 16 h by air oven (Binder, Tuttlingen, Germany). Ash content was determined by incineration 525 °C for 4 h in muffle furnace (Furnace Carbolite SN. 80 16919). Fat content was measured by the difference in weight before and after petroleum ether extraction by using an extraction vessel (ANKOM, Macedon, NY, USA). Non-digestible carbohydrates (total crude fibre) after acidic digestion in H_2_SO_4_ solution at 90 °C followed by alkali digestion in NaOH solution at 90 °C by using fiber analyzer (ANKOM, Macedon, NY, USA). The remained weight was considered as crude fiber to determine fiber content. Kjeldahl method was used to determine protein content based on nitrogen quantification.

### 2.3. Color Measurement

The changes in color traits (L*a*b*) was measured by Minolta Chroma Meter CR-400 reflectance colorimeter according to the Commission Internationale de l’Eclairage (CIE) system [17]. The system consists of three dimensions: one for luminance (L*-lightness) and two for color (a*-green to red; b*-blue to yellow). The instrument was calibrated against standard white ceramic tile (Y = 93.9, x = 0.3130 and y = 0.3190). From each group, four measurements were taken from four different areas on vacuum packages. Color indices were measured in triplets and the mean value was reported. 

### 2.4. Macro and Microscope Examination

The photos for *O. syriacum* leaves were captured by M190C3JG camera with a resolution of 48 MP and Samsung Galaxy A10s camera with a resolution of 13 MP Pictures to evaluate the morphological changes due to freeze-drying and solar drying in comparison to fresh leaves. 

Microscopic pictures for fresh, freeze-, and solar dried leaves were captured by microscope supplied with camera OMAX M8327L-C50V3 at 10×. Pictures were adjusted by OMAX ToupView computer application.

### 2.5. Microbiological Analysis

Ten g of dried leaves, obtained by the different drying methods, were mixed with 90 mL of peptone water in a stomacher bag, then the sample was homogenized in a STOMACHER^®^400 CIRCULATOR for 2 min at 260 RPM. Plate Count Agar (PCA) medium was used for bacterial growth to count the total viable bacterial at 37 °C. Sabouraud Dextrose Agar (SDA) medium was used to count total number of yeast and mold. Incubation was performed at room temperature for 5 days.

### 2.6. Sensory Analysis

Hedonic scale was used to evaluate the several sensory traits such as color (greenness, yellowness, acceptance), odor (dried grass, woody, acceptance), freshness, taste acceptance, earthy aroma, flavor acceptance, and overall acceptance. Eleven points hedonic scale from 0 to 10, where 0 = disliked extremely/weak, 5 = neither liked nor disliked/neutral, and 10 = liked extremely/strong. Triangle test was employed to differentiate between freeze- and solar dried *O. syriacum* samples. The freeze- and solar dried *O. syriacum* were ground and offered for assessors. 41 assessors from both participated in sensory analysis

### 2.7. Statistical Analysis

One-way ANOVA test has been carried out to find the effect of drying techniques on proximate chemical, color, and microbiological analyses data by using SPSS software. Moreover, two-way ANOVA test was used to evaluate the effect of drying techniques and storage period on microbiological analysis. Duncan multiple range test was used to separate the means and (*p* ≤ 0.05) was considered as significant. 

## 3. Results and Discussion

### 3.1. Chemical Analysis of Za’atar

The results of the proximate chemical composition (moisture, protein, fat, fiber, ash, total carbohydrates) was shown in Table 2 (based on wet basis) and in Table 3 (based on dry basis). The result of moisture content was in agreement with previous studies. In general, the moisture content of *O. syriacum* is usually affected by weather conditions, type of species, age of croup, and fertility of soils. Balladin and Headley [18] found that the moisture content of fresh thyme was 75.12%. In addition, Doymaz [19] and Sárosi et al. [20] found similar results.

The result of moisture content obtained in our study was a little bit higher than results found in the literature and this is probably due to the differences in farming conditions. The moisture content was dramatically reduced after solar and freeze drying if compared to fresh *O. syriacum* (12.59 and 13.2 vs. 81.3%, *p* < 0.05), respectively. It was found that moisture content of fresh thyme was reduced from 52.25% to 4.66% and 2.30% after solar- and freeze drying, respectively [21]. Our study showed that there was no significant difference in moisture reduction between solar and freeze drying.

For fat content, it was found that there were no significant differences between fat content in fresh and freeze-dried *O. syriacum* while solar dried *O. syriacum* exhibited significantly lower fat content than fresh and freeze-dried *O. syriacum*. These results may be attributed due to volatilization of essential oils during solar drying. Pirbalouti et al. [22] revealed that the essential oil yield in basil was reduced after solar drying. In general, our findings were in agreement with previous studies in the context of the effect of freeze drying on essential oil yield in comparison to solar. Freeze dried herbs exhibited higher essential oil yield than solar dried herbs [21,23]. In addition, Portillo-Estrada et al. [24] found that freeze drying exhibited higher retention for volatiles compounds than air-drying at different temperature.

There were no significant differences in protein and ash contents between fresh, freeze-, or solar dried *O. syriacum* while there were significant differences in fiber and total carbohydrate contents based on a dry basis between dried *O. syriacum* groups and the fresh one. These results may be attribute to determination of carbohydrates by differences. Moreover, the significant difference in moisture levels between fresh and dried za’atar, may influence fiber and total carbohydrate levels in *O. syriacum*. 

There were no significant differences in ash content between freeze dry- and solar dry *O. syriacum*. The results of ash content obtained in this study was quite similar to previous study. It was found that ash content in thyme fresh was 1.60%, after solar drying the ash content was increased due to higher water content in the fresh sample [18].

### 3.2. Color Measurements

**Figure 1:**
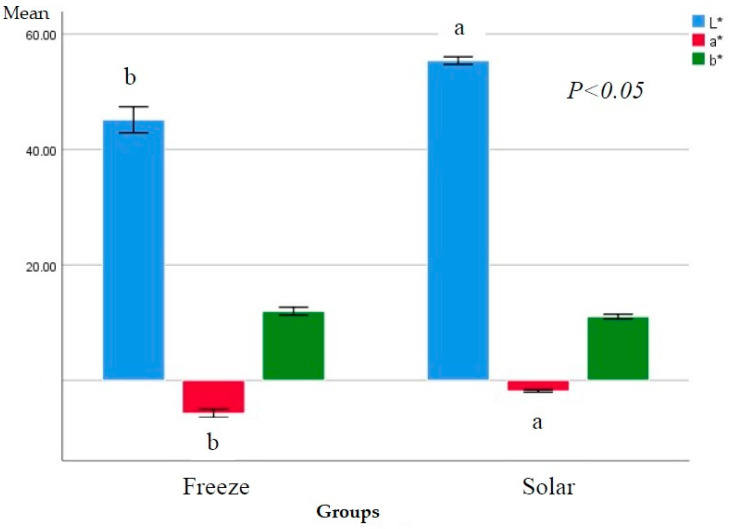
Color traits (L*a*b*) of freeze- and solar- dried *O. syriacum.* Different superscript letters for the same color index of freeze- and solar- dried *O. syriacum* are significantly different (*p* ≤ 0.05).

**Figure 2:**
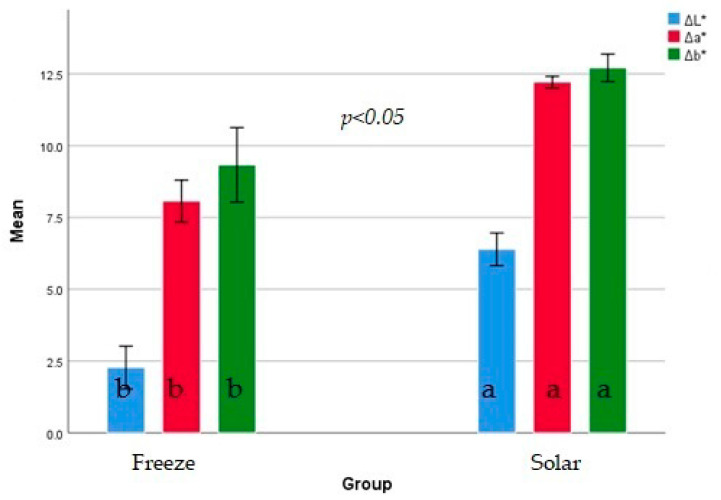
The change in color traits (ΔL*, Δa*, Δb*) calculated by the differences in the values before and after freeze- and solar- dried *O. syriacum.* Different superscript letters for the same color index of freeze- and solar- dried *O. syriacum* are significantly different (*p* ≤ 0.05).

The effect of freeze and solar drying on color traits (L*a*b*) was shown in Figure 1 and the change in color after drying in comparison to fresh was shown in Figure 2. Freeze dried *O. syriacum* had significantly lower L*-value and a*-value than solar dried *O. syriacum*. There was no significant difference in b*-values between freeze- and solar- dried *O. syriacum*. From a*-value, freeze dried *O. syriacum* exhibited higher degree of greenness from solar dried *O. syriacum*. Moreover, solar dried *O. syriacum* was lighter in color. Doymaz et al. [19] found that b* values of freeze-dried thyme did not significantly differ from solar dried thyme. Rahimmalek & Goli [21] found that freeze-dried thyme had the lower a*-values than solar dried thyme. 

It was found that the changes in color index (L*a*b) of solar-dried *O. syriacum* was higher than in freeze-dried *O. syriacum* in respect to fresh. This indicates the destruction rate of natural pigment by freeze drying was lower than solar drying. In this context, it was found that freeze-drying exhibited higher protection chlorophyll than solar drying [25].

### 3.3. Macro and Microscopic Evaluation of Structure

**Figure 3:**
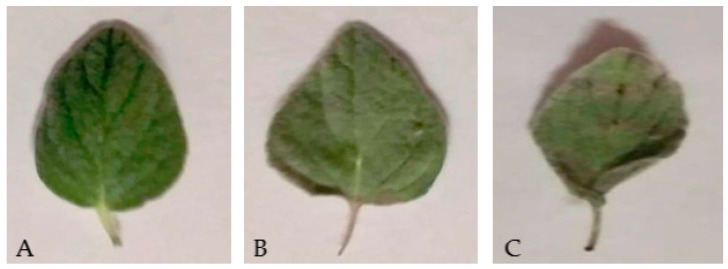
The morphological changes in *O. syriacum* leaves due to drying, (**A**) fresh, (**B**) freeze-dried, (**C**) solar dried.

The effect of drying techniques on the morphological changes of *O. syriacum* leaves was shown in Figure 3. The photos showed clearly that there were changes in the morphology of *O. syriacum* leaves after drying in comparison to fresh leaves. Solar dried leaves showed more darken areas and less fresh green color than freeze dried leaves. During solar drying, leaves are more expose to enzymatic browning reactions. The enzymatic browning reactions occur between oxygen, polyphenol oxidase (PPO) enzymes, and phenolic compounds. Enzymatic browning reactions continue during solar drying while they stop during freeze drying [26]. Another cause for browning areas in solar dried leaves is Maillard reaction that induced by heating. It was found that Maillard reaction increased during drying of thyme at high temperature [27]. Freeze drying techniques imparted better visual appearance for dill than other drying techniques [28].

**Figure 4:**
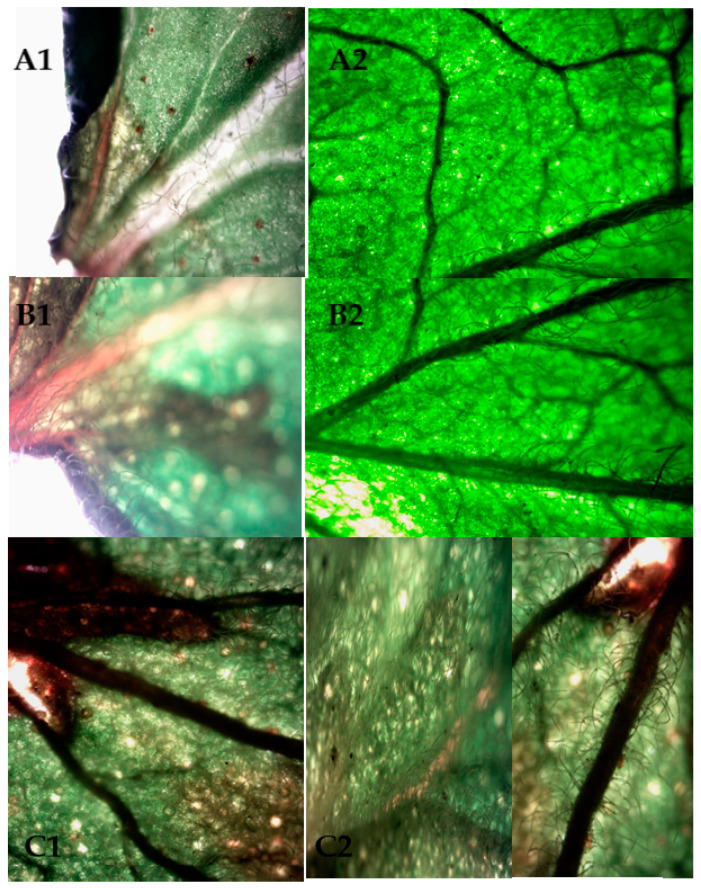
Microscopic pictures of za’atar leaves, (**A1**) fresh image near leave stem, (**A2**) fresh image near leave center, (**B1**) freeze-dried leave near stem, (**B2**) freeze-dried leave center, (**C1**) solar dried leave near stem, (**C2**) solar dried near center.

Figure 4 showed the effect of drying techniques on the microstructures of leaves by using light microscope. Drying caused loss of structural integrity as well as leave hairs. This damage was more severe in solar drying if compared to freeze drying. The edges of fresh and freeze-dried leaves were quite intact while the curvature edge shape of solar dried leaves was lost. Díaz-Maroto et al. [29] used SEM to compare the effect of freeze drying and air drying on the microstructure of spearmint. The study showed the structural damage for epithelial cells in freeze-drying was less in comparison to air oven drying. Klungboonkrong et al. [30] found that SEM microstructure pictures of java tea leaves revealed that freeze-dried leaves exhibited less damage in cellular structure than other drying technologies.

### 3.4. Microbiological Analysis of Origanum syriacum

The results of microbiological analysis for fresh, freeze-, and solar-dried *O. syriacum* was shown in Table 4. Both solar and freeze-drying techniques reduced the total aerobic bacterial count significantly if compared to fresh *O. syriacum*. Our findings were in agreement with previous studies [31,32,33].

This can be attribute to the fact that removal of moisture makes disturbance and destruction for cell wall as well as for DNA [34]. Freeze-dried *O. syriacum* exhibited significantly the lowest total plate count (TPC) while fresh za’atar had the highest count at the beginning of storage. After 30 days of storage (room temperature), the TAC of freeze-dried *O. syriacum* increased by 1 Log while solar dried exhibited slight reduction in TAC. Solar drying was less effective in reduction of total aerobic bacteria and fungi than freeze drying. Freeze-dried *O. syriacum* had significantly lower total aerobic count (4.92 vs. 5.23, *p* < 0.05) than solar-dried *O. syriacum* at the beginning of storage (Day 1). Moreover, freeze-dried *O. syriacum* exhibited significantly lower yeasts and mold count (4.59 vs. 5.36, *p* < 0.05) than solar-dried *O. syriacum* at the beginning of storage (Day 1). The total yeasts and molds count increased significantly (4.18 vs. 5.36, *p* < 0.05) after solar drying if compared to before drying (fresh). This result can be attributed due to the contamination by air. It was found that sun-dried ginger had higher content of total bacteria and this was attributed due to contaminants from the surrounded environment [35]. Dereje and Abera [33] found that solar dried mango exhibited higher content fungal count than freeze dried mango. Malmsten et al. [32] revealed that freeze-drying was more effective in lowering yeast and mold count than air-drying.

After 30 days of storage at room temperature, the total aerobic count of freeze-dried *O. syriacum* had significantly increased while the yeasts and molds count did not significantly change. Solar-dried *O. syriacum* did exhibit any significant change in total aerobic and fungi count after 30 days of storage if compared to the beginning.

### 3.5. Sensory Analysis of Origanum syriacum 

Triangle test showed that 27 out of 41 panelists were able to distinguish the odd sample that was a freeze-dried sample from the other two solar dried samples. The panelists were significantly able to differentiate between freeze- and solar dried za’atar. There were significant differences (25 out of 41 panelists at *p* < 0.001, 22 out of 41 panelists at *p* < 0.01, 20 out of 41 panelists at *p* < 0.05, and 19 out of 41 panelists at *p* < 0.1). The study showed that main discriminators in triangle test were odor, color, and appearance. About 41% of panelists identified the odd sample by both color and odor, 11% of panelists distinguished odd sample by odor and appearance, 33% of panelists distinguished odd by odor only, and the remaining part of panelists distinguish odd by color.

**Figure 5:**
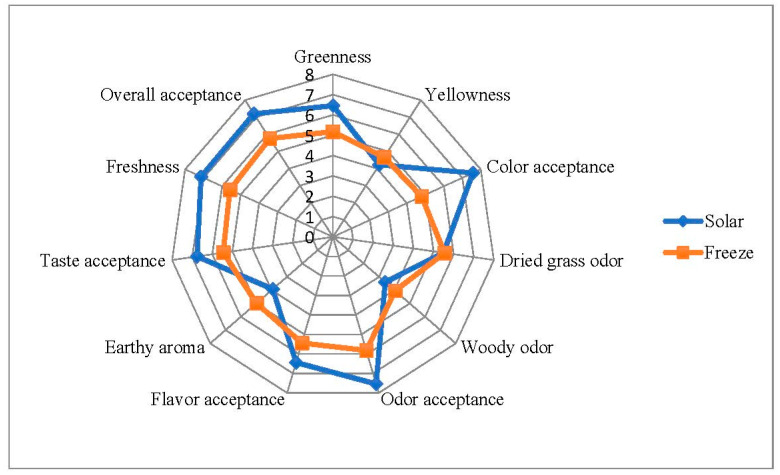
Mean score of sensory analysis for freeze-and solar-dried *O. syriacum* based on 11-point hedonic scale.

The results of sensory analysis by the hedonic scale for freeze- and solar dried za’atar was shown in Figure 5. It was found freeze-dried za’atar exhibited significantly lower score of color, odor, taste, and overall acceptance than solar dried za’atar. Our study showed that panelists preferred the flavor of solar dried za’atar than freeze-dried za’atar. The degree of greenness of solar dried za’atar was significantly higher than freeze-dried za’atar and this result supported by b* values. There were no significant differences between solar- and freeze-dried za’atar in dried grass, woody odors and earthy aroma. Solar dried za’atar exhibited significantly higher degree of freshness than freeze-dried za’atar. This may be due to the higher overall acceptance of solar died za’atar as well as the Amadori compounds (the precursors for many aromas and flavors) that generated by Maillard reaction [36]. There are no available data about the differences in sensory traits between solar and freeze- dried za’atar. Some studies compared between freeze drying and air drying as well as microwave. Mudalal and Abu-Khalaf [37] found that electronic nose was able to differentiate between freeze- and solar-dried *O. syriacum*. This study indicated that freeze- and solar-dried *O. syriacum* had different profile of flavoring compounds which may affect the sensory traits. Freeze-dried spearmint exhibited stronger spice aroma than air-dried spearmint [29]. Calín-Sánchez et al. [38] showed that freeze-dried thyme exhibited more preferred sensory attributes that convective and vacuum-microwave dried thyme. Similar findings were observed by Sárosi et al. [20]. Atalla et al. [7] found that freeze dried *O. syriacum* had mustier and earthy aroma while air dried *O. syriacum* exhibited stronger aroma and flavors than freeze dried *O. syriacum*.

In conclusion, freeze-drying achieved a moisture level reduction similar to solar drying. However, freeze-drying was better than solar drying in preserving the fat content of za’atar. Additionally, freeze-dried imparted more fresh appearance (in respect to color) for za’atar than solar drying. Freeze drying technique was effective in preservation of the morphological features and keeping low microbial level of *O. syriacum* leaves. Solar-dried *O. syriacum* had higher sensory preferences for consumers in comparison to freeze drying. 

## Figures and Tables

**Table 1:** Weather conditions during solar drying ([15], accessed on 15 September 2020).

Date	Temperature °C	Humidity %	Barometer atm	Wind mph
9 August 2020	31/29	56	0.994	13.671
10 August 2020	31/30	54	0.995	14.914
11 August 2020	31/29	57	0.996	13.671
12 August 2020	31/30	58	0.994	12.428
13 August 2020	32/30	58	0.993	11.185
14 August 2020	32/30	59	0.992	11.185
15 August 2020	32/30	60	0.991	11.185

**Table 2:** Proximate chemical composition percentages based on wet basis for fresh, solar, and freeze-dried *O. syriacum*.

	Fresh Mean ± STD	Solar Mean ± STD	Freeze Mean ± STD	*p*-Value
Moisture%	81.32 ± 0.83 ^a^	13.20 ± 0.29 ^b^	12.59 ± 2.05 ^b^	<0.05
Protein%	2.08 ± 0.30	1.81 ± 0.69	2.04 ± 0.31	0.636
Fat%	1.82 ± 0.32 ^a^	1.10 ± 0.06 ^b^	1.64 ± 0.07 ^a^	<0.05
Ash%	2.11 ± 0.12	2.12 ± 0.03	2.16 ± 0.09	0.735
Fiber%	4.13 ± 0.28	4.37 ± 0.16	4.38 ± 0.23	0.205
Total carbohydrates%	8.78 ± 0.63	9.52 ± 0.79	8.70 ± 0.28	0.146

Different superscript letters in a row are significantly different (*p* ≤ 0.05).

**Table 3:** Proximate chemical composition percentages based on dry basis for fresh, solar, and freeze-dried *O. syriacum*.

	Fresh Mean ± STD	Solar Mean ± STD	Freeze Mean ± STD	*p*-Value
Protein%	11.05 ± 1.79	10.03 ± 3.08	10.78 ± 1.65	0.747
Fat%	9.61 ± 1.76 ^a^	6.28 ± 0.79 ^b^	8.47 ± 0.0.47 ^a^	<0.05
Ash%	11.17 ± 0.35	11.18 ± 0.16	11.50 ± 0.58	0.306
Fiber%	21.81 ± 0.10 ^b^	22.95 ± 0.68 ^a^	23.33 ± 0.98 ^a^	<0.05
Total carbohydrate%	46.36 ± 1.27 ^b^	50.31 ± 4.18 ^a^	45.98 ± 1.48 ^b^	0.05

Different superscript letters in a row are significantly different (*p* ≤ 0.05).

**Table 4:** Microbiological analysis for fresh, freeze-, and solar-dried *O. syriacum*.

	Fresh Mean ^1^ ± STD	Solar Mean ± STD	Freeze Mean ± STD	*p*-Value
TPC day 1	5.98 ± 0.12 ^a^	5.23 ± 0.23 ^b^	4.92 ± 0.05 ^c^	<0.05
TPC day 30	-	4.84 ± 0.59 ^b^	5.84 ± 0.03 ^a^	<0.05
Yeast and mold day 1	4.18 ± 0.62 ^b^	5.36 ± 0.34 ^a^	4.59 ± 0.13 ^b^	<0.05
Yeast and mold day 30	-	5.01 ± 0.46	4.41± 0.35	0.254

Different superscript letters in a row are significantly different (*p* ≤ 0.05). ^1^ Mean value was presented in log CFU/g (CFU colony forming unit).

## Data Availability

Not applicable.

## References

[1] Amadio C., Medina R., Dediol C., Zimmermann M.É., Miralles S. (2011). Oregano essential oil: A potential food additive. Rev. Fac. Cienc. Agrar..

[2] Cosge B., Turker A., Ipek A., Gurbuz B., Arslan N. (2009). Chemical compositions and antibacterial activities of the essential oils from aerial parts and corollas of *Origanum acutidens* (Hand.-Mazz.) Ietswaart, an endemic species to turkey. Molecules.

[3] Meyers M. (2005). Oregano and Marjoram: An Herb Society of America Guide to the Genus Origanum.

[4] Vernin G., Lageot C., Gaydou E.M., Parkanyi C. (2001). Analysis of the essential oil of *Lippia graveolens* HBK from el salvador. Flavour Fragr. J..

[5] Avila-Sosa R., Gastélum-Franco M., Camacho-Dávila A., Torres-Muñoz J., Nevárez Moorillón G. (2010). Extracts of mexican oregano (*Lippia berlandieri* Schauer) with antioxidant and antimicrobial activity. Food Bioprocess Technol..

[6] United Nations-Economic and Social Commission for Western Asia (2010). Best Practices and Tools for Increasing Productivity and Competitiveness in the Production Sectors: Assessment of Zaatar Productivity and Competitiveness in Lebanon. https://www.unescwa.org/sites/www.unescwa.org/files/publications/files/sdpd-10-tp3.pdf.

[7] Atallah S.S., Saliby I.E., Baalbaki R., Talhouk S.N. (2010). Effects of different irrigation, drying and production scenarios on the productivity, postharvest quality and economic feasibility of *Origanum syriacum*, a species typically over-collected from the wild in Lebanon. J. Sci. Food Agric..

[8] Wakim L.H., Beyrouthy M.E., Mnif W., Dhifi W., Salman M., Bassal A. (2013). Influence of drying conditions on the quality of *Origanum syriacum* L.. Nat. Prod. Res..

[9] Alwafa R., Mudalal S., Mauriello G. (2021). *Origanum syriacum* L. (Za’atar), from Raw to Go: A Review. Plants.

[10] Udomkun P., Romuli S., Schock S., Mahayothee B., Sartas M., Wossen T., Njukwe E., Vanlauwe B., Müller J. (2020). Review of solar dryers for agricultural products in Asia and Africa: An innovation landscape approach. J. Environ. Manag..

[11] Hossain M., Barry-Ryan C., Martin-Diana A., Brunton N. (2010). Effect of drying method on the antioxidant capacity of six Lamiaceae herbs. Food Chem..

[12] Abu-Lafi S., Odeh I., Dewik H., Qabajah M., Imam A., Dembitsky V.M., Hanus L.O. (2007). Natural compounds of Palestine flora. Comparison analysis by static headspace and steam distillation GC-MS of semivolatile secondary metabolites from leaves of cultivated Palestinian Majorana syriaca. Biomed. Pap. Med. Fac. Palacky Univ. Olomouc.

[13] Awada F., Kobaissi A., Chokr A., Hamze K., Hayar S., Mortada A. (2012). Factors affecting quantitative and qualitative variation of thyme (*Origanum syriacum* L.) essential oil in Lebanon. Adv. Environ. Biol..

[14] Shehadeh M., Jaradat N., Al-Masri M., Zaid A., Hussein F., Khasati A., Suaifan G., Darwish R. (2019). Rapid, cost-effective and organic solvent-free production of biologically active essential oil from Mediterranean wild *Origanum syriacum*. Saudi Pharm. J..

[15] Timeanddate. https://www.timeanddate.com/weather/palestine/tulkarm/historic?month=8&year=2020.

[16] AOAC (1990). Association of Official Analytical Chemists.

[17] Luo M.R., Luo R. (2015). CIELAB. Encyclopedia of Color Science and Technology.

[18] Balladin D.A., Headley O. (1999). Evaluation of solar dried thyme (*Thymus vulgaris* Linné) herbs. Renew. Energy.

[19] Doymaz I. (2011). Drying of thyme (*Thymus Vulgaris* L.) and selection of a suitable thin-layer drying model. J. Food Process. Preserv..

[20] Sárosi S., Sipos L., Kókai Z., Pluhár Z., Szilvássy B., Novák I. (2013). Effect of different drying techniques on the aroma profile of Thymus vulgaris analyzed by GC-MS and sensory profile methods. Ind. Crops Prod..

[21] Rahimmalek M., Goli S.A.H. (2013). Evaluation of six drying treatments with respect to essential oil yield, composition and color characteristics of Thymys daenensis subsp. daenensis. Celak leaves. Ind. Crops Prod..

[22] Pirbalouti A.G., Mahdad E., Craker L. (2013). Effects of drying methods on qualitative and quantitative properties of essential oil of two basil landraces. Food Chem..

[23] Mirhosseini F., Rahimmalek M., Pirbalouti A.G., Taghipoor M. (2015). Effect of different drying treatments on essential oil yield, composition and color characteristics of Kelussia odoratissima Mozaff. J. Essent. Oil Res..

[24] Portillo-Estrada M., Copolovici L., Niinemets Ü. (2015). Bias in leaf dry mass estimation after oven-drying isoprenoid-storing leaves. Trees.

[25] Lafeuille J.L., Lefèvre S., Lebuhotel J. (2014). Quantitation of chlorophylls and 22 of their colored degradation products in culinary aromatic herbs by HPLC-DAD-MS and correlation with color changes during the dehydration process. J. Agric. Food Chem..

[26] Singh B., Suri K., Shevkani K., Kaur A., Kaur A., Singh N. (2018). Enzymatic browning of fruit and vegetables: A review. Enzym. Food Technol. Improv. Innov..

[27] Zhang C., Lu J., Wang Y., Ma Y., Zhao X. (2015). Effect of drying temperature on sensory and flavor of thyme. Ic3me.

[28] Raghavan B., Abraham K.O., Shankaranarayana M.L., Koller W.D. (1994). Studies on Flavor Changes During Drying of Dill (*Anethum sowa* Roxb.) Leaves. J. Food Qual..

[29] Díaz-Maroto M.C., Pérez-Coello M.S., Viñas M.A.G., Cabezudo M.D. (2003). Influence of drying on the flavor quality of spearmint (*Mentha spicata* L.). J. Agric. Food Chem..

[30] Klungboonkrong V., Phoungchandang S., Lamsal B. (2018). Drying of Orthosiphon aristatus leaves: Mathematical modeling, drying characteristics, and quality aspects. Chem. Eng. Commun..

[31] Deans S.G., Svoboda K.P., Bartlett M.C. (1991). Effect of microwave oven and warm-air drying on the microflora and volatile oil profile of culinary herbs. J. Essent. Oil Res..

[32] Malmsten T., Pääkkönen K., Hyvönen L. (1991). Packaging and Storage Effects on Microbiological Quality of Dried Herbs. J. Food Sci..

[33] Dereje B., Abera S. (2020). Effect of some pretreatments before drying on microbial load and sensory acceptability of dried mango slices during storage periods. Cogent Food Agric..

[34] Bourdoux S., Li D., Rajkovic A., Devlieghere F., Uyttendaele M. (2016). Performance of Drying Technologies to Ensure Microbial Safety of Dried Fruits and Vegetables. Compr. Rev. Food Sci Food Saf..

[35] Eze J., Agbo K. (2011). Comparative studies of sun and solar drying of peeled and unpeeled ginger. Am. J. Sci. Ind. Res..

[36] Cardelle-Cobas A., Moreno F.J., Corzo N., Olano A., Villamiel M. (2005). Assessment of initial stages of Maillard reaction in dehydrated onion and garlic samples. J. Agric. Food Chem..

[37] Mudalal S., Abu-Khalaf N. (2021). Electronic nose to differentiate between several drying techniques for *Origanum syriacum* leaves. Food Res..

[38] Calín-Sánchez Á., Figiel A., Lech K., Szumny A., Carbonell-Barrachina Á (2013). Effects of Drying Methods on the Composition of Thyme (*Thymus vulgaris* L.) Essential Oil. Dry Technol..

